# Incorporation of Plant-Based Diet Surpasses Current Standards in Therapeutic Outcomes in Inflammatory Bowel Disease

**DOI:** 10.3390/metabo13030332

**Published:** 2023-02-23

**Authors:** Mitsuro Chiba, Norikazu Morita

**Affiliations:** 1 Division of Gastroenterology, Akita City Hospital, Akita 010-0933, Japan; 2 Morita GI Clinic, Fukuoka 810-0041, Japan

**Keywords:** Crohn’s disease, diet, inflammatory bowel disease, natural course, plant-based diet, relapse, semi-vegetarian diet, therapy, ulcerative colitis

## Abstract

There has been no study of the therapeutic effect of a plant-based diet (PBD) in inflammatory bowel disease (IBD) except for our studies in Japan. In this review, we describe the rationale for the requirement of PBD in IBD and the outcomes of our modality incorporating PBD together with a literature review. The biggest problem in current therapy for IBD is the lack of a widely appreciated ubiquitous environmental factor in IBD. Therefore, a radical strategy against IBD has not been established. Japanese data showed an increased incidence of IBD in association with dietary westernization. Current global consumption consists of an excess of unhealthy foods and a shortage of healthy foods recognized as pro-inflammatory. Patients with IBD are no exception. One of the recommended healthy reference diets is PBD recognized as anti-inflammatory. We assert that IBD occurs in susceptible individuals mainly as a result of our omnivorous (westernized) diet. Therefore, we developed and began to provide a PBD, a lacto-ovo-vegetarian diet, for IBD patients in 2003. Infliximab and PBD as first-line (IPF) therapy was administered for all patients with newly developed Crohn’s disease (CD) and for severe ulcerative colitis (UC). Our modality broke the barrier of primary nonresponders to biologics, with a remission rate of 96% in CD, and created a new relapse-free course in slightly over half of the patients (52%) with CD. Based on the rationale derived from available evidence and the clinical outcomes, PBD is highly recommended for IBD.

## 1. Introduction 

The incidence of inflammatory bowel disease (IBD), a collective term for Crohn’s disease (CD) and ulcerative colitis (UC), has been increasing over time and expanding to different regions around the world, indicating that IBD is a global disease [1]. IBD is a polygenic disease thought to be triggered by environmental factors. It is accepted that the westernization of lifestyle is a major driver of a growing incidence of IBD and that IBD is a dysregulated mucosal inflammation of gut microbiota. There are many review articles on environmental factors in IBD, but none refers to which factor contributes most to triggering the onset of IBD [2]. Identification and recognition of the greatest environmental factor are a prerequisite for treating and preventing the disease [3]. The biggest problem in current therapy for IBD is the lack of a widely appreciated key (ubiquitous) environmental factor in a westernized lifestyle in IBD. We assert that IBD occurs in susceptible individuals mainly due to our omnivorous (westernized) diet [3,4]. Therefore, we developed and began to provide a plant-based diet (PBD), a lacto-ovo-vegetarian diet, for IBD patients in 2003 to counter an omnivorous (westernized) diet [5]. We achieved far better outcomes in both UC and CD in both the induction and quiescent phases compared to the current standard therapy. There has been no study of the therapeutic effect of PBD in IBD except for our prospective studies in Japan. In the first half of this review, we describe the rationale for the requirement of PBD in IBD. In the last half, we describe the outcomes of our modality incorporating PBD together with a review of the literature. Infliximab and PBD as first-line (IPF) therapy broke the barrier of primary nonresponders to biologics (around 30%) and created a new relapse-free course in CD. This review will directly answer two questions on diet and, indirectly, seven questions among the top 10 research questions in the treatment of IBD raised in 2017 (Table 1) [6]. Needless to say, this will answer the most common question asked by patients: “What should I eat?” [7,8].

## 2. Our Current Westernized Diet Is the Ubiquitous Environmental Factor in IBD

Our current westernized diet is problematic and is most likely the ubiquitous environmental factor in IBD. This concept forms the rationale for replacing the current diet with adequate diets, most likely PBD.

### 2.1. Bitter Experiences with Ordinary Food in Crohn’s Disease

**Figure 1:**
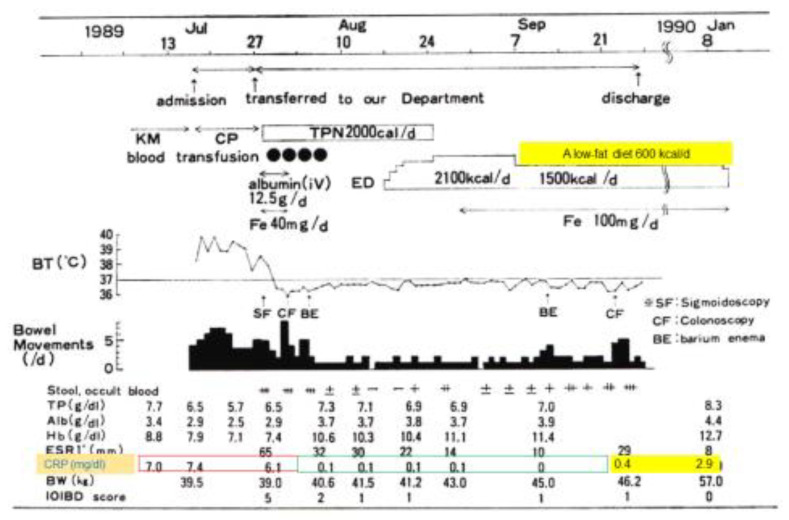
Clinical course of a 14-year-old girl with an initial attack of Crohn’s disease. KM, kanamycin; CP, chloramphenicol; TPN, total parenteral nutrition; ED, elemental diet; Fe, ferrum; BT, body temperature; TP, total protein; Alb, albumin; Hb, hemoglobin; ESR, erythrocyte sedimentation rate; CRP, C-reactive protein (reference ≤0.3 mg/dL); BW, body weight; IOIBD score, International Organization for Inflammatory Bowel Disease score.

In 1989, the incidence of IBD was increasing in Japan. Physicians observed amelioration of fever, diarrhea, abdominal pain, and poor appetite associated with CD with the use of total parenteral nutrition. Parenteral nutrition was then gradually replaced with hospital meals. Even with nutritionally balanced hospital meals, C-reactive protein (CRP) increased above the reference range shortly after switching. Both physicians and their patients thought meals per se caused gut inflammation [5]. An elemental diet (ED) (amino acid-based) was also used for CD. In the case in Figure 1, a 14-year-old girl was treated with total parenteral nutrition and then enteral nutrition as ED, followed by home elemental enteral hyperalimentation (HEEH). She received a low-fat diet (600 kcal/day) along with a reduction of ED from 2100 to 1500 kcal/day. At the time of discharge, she was clinically in remission. CRP, however, became abnormal. It further increased to 2.9 mg/dL 3.5 months after discharge (Figure 1) [9]. Abnormal CRP is well-known as a sign of future relapse [10,11].

**Figure 2:**
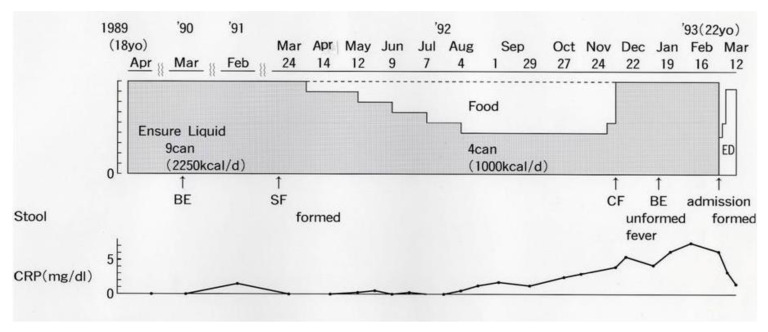
An attempt to gradually replace Ensure Liquid (polymeric enteral nutrition) with ordinary food in a male student with Crohn’s disease. BE, barium enema; SF, sigmoido (fiber)scopy; CF, colono (fiber)scopy; ED, elemental diet; CRP, C-reactive protein (reference ≤0.3 mg/dL).

An 18-year-old male student moved to Akita City to start university life (Figure 2). He attained remission with total ED, which was replaced by a polymeric (whole protein based) enteral diet, Ensure Liquid (Meiji Milk Product Co. Ltd., Tokyo, Japan). He had been in remission for 3 years with nine cans/day of Ensure Liquid (250 kcal, 250 mL/can) without any ordinary foods or medication. One year before graduation, he wanted to replace Ensure Liquid with ordinary food in preparation for becoming a member of society. The gradual replacement of Ensure Liquid with ordinary food, i.e., a decrease of one can/day every 4 weeks, was undertaken. The replacement was unsuccessful due to a relapse after withdrawing five cans/day of Ensure Liquid (Figure 2) [12]. 

These experiences led to a partial replacement of ordinary food with elemental or polymeric enteral nutrition. Consequently, previous Japanese guidelines for quiescent CD incorporated an elemental diet in addition to regular meals [13]. Therefore, it is apparent that meals should be replaced with an adequate diet in CD. On the other hand, a different response to hospital meals is observed in UC, i.e., they make recovery smooth. This suggests that an adequate diet is critically needed in CD, more so than in UC.

### 2.2. Environmental Factors in IBD 

There have been many studies on environmental factors in IBD. Representative environmental factors are as follows: smoking, breastfeeding, non-steroidal anti-inflammatory drugs, antibiotic use in childhood, oral contraceptives, appendectomy, air pollution, and diet [2]. There are several conditions for a ubiquitous environmental factor in IBD [4]. Considering that the epidemiology of IBD is similar in both CD and UC, the ubiquitous environmental factor in IBD should not be divergent between UC and CD but should be common to both UC and CD [4,14]. The factor should not differ among geographical areas. The other consideration is dysbiosis of gut microbiota because it is universally found in IBD [15]. Therefore, the factor can influence gut microbiota. Last, the majority of IBD patients should be exposed to the factor. We conclude that diet is the most ubiquitous environmental factor in IBD (Table 2) [4].

### 2.3. Epidemiologic Studies of the Relation between Diets and IBD

#### 2.3.1. Risky or Preventive Foods for IBD

There might be a great variety of dietary transitions (westernization) in terms of speed, degree, and mode of adaption to the transition among countries, races, and individuals based on their culture and traditions [14]. Other lifestyles associated with socioeconomic transition and related to IBD are likewise heterogeneous. Therefore, a cautious comprehensive analysis is needed to interpret the results on the relation between diets and IBD. This seems to be one of the explanations for inconsistent results or contradictory findings with respect to the relationship between diet and IBD [16,17,18,19,20,21]. The lack of consistent evidence has hampered the formulation of dietary guidelines for IBD, with the exception of exclusive enteral nutrition in CD [22]. Recently, however, risk factors in IBD have been summarized as follows: not eating enough vegetables and fruits and excess consumption of animal fat, animal protein, and sugar [16,17,18,19,20,21].

**Figure 3:**
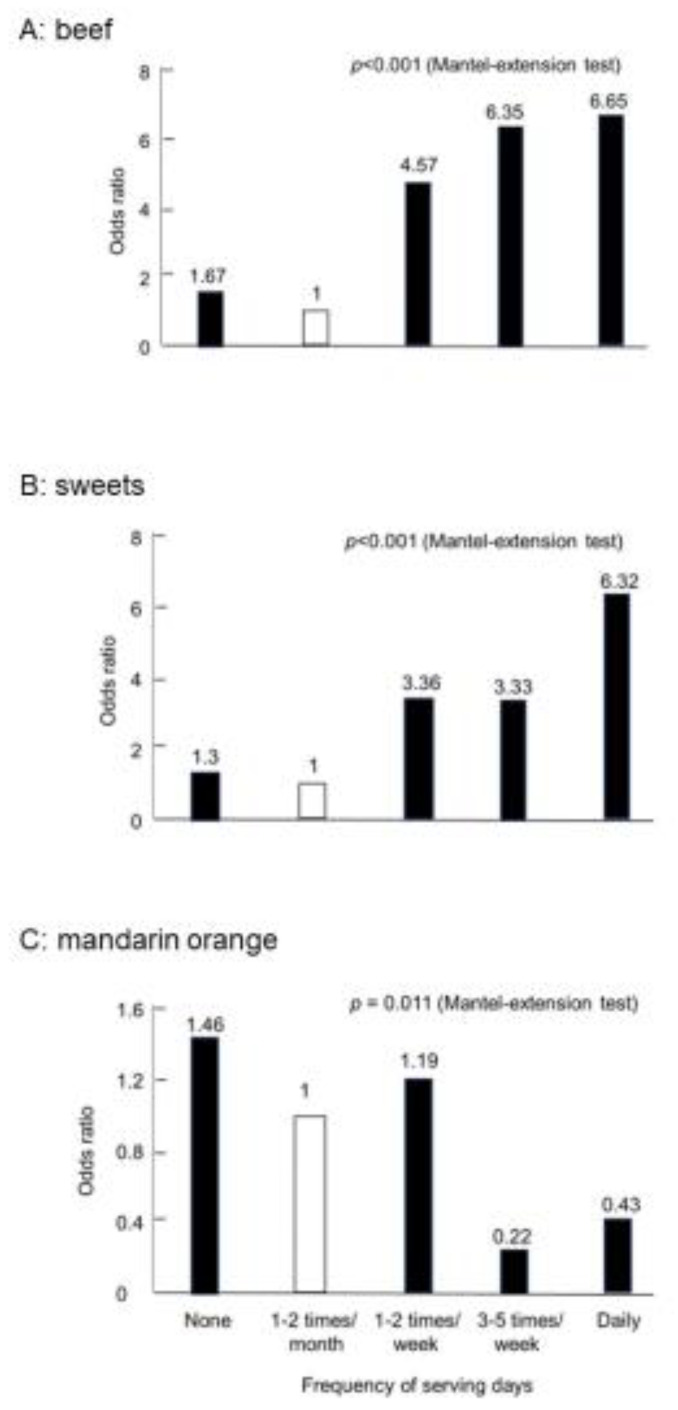
Case-control study in Crohn’s disease in Japan (CD *n* = 104).

We also obtained similar results in Japanese studies. The Research Committee of Epidemiology of Intractable Diseases, the Ministry of Health and Welfare, Japan, conducted case-control studies on IBD patients [23,24]. A self-administered questionnaire was used to obtain information on pre-illness diet from newly diagnosed IBD patients. Pooled healthy controls were used for matching for sex, age, and study areas. Odds ratios were obtained with a conditional logistic model, and correlations were analyzed using the Mantel extension test. Thirty-seven foods studied are distributed into four categories: risky foods for both CD and UC, preventive foods for both, foods neither risky nor preventive for both, and risky or preventive foods for either disease (Table 3). None of the foods showed opposite reactions, such as being high risk for CD but preventive for UC and vice versa. Beef was a high-risk food in CD (Figure 3A) but not in UC (Table 3). In addition, sweets were a high-risk food in both (Figure 3B). However, mandarin oranges were a preventive food for both (Figure 3C). On the whole, western foods such as beef, cheese, and sweets were risky foods, whereas traditional Japanese foods such as edible wild plants, pickles, and green tea were prophylactic foods. Eggs and milk were not high-risk foods in these studies (Table 3). Yogurt was listed as a high-risk food for both CD and UC in these studies [23,24]. We think that yogurt was listed because it was mainly served along with western foods. Plain yogurt is a probiotic. Therefore, we recommend yogurt to patients with IBD, as described later.

#### 2.3.2. Dietary Transition from a Traditional Diet to a Westernized Diet

**Figure 4:**
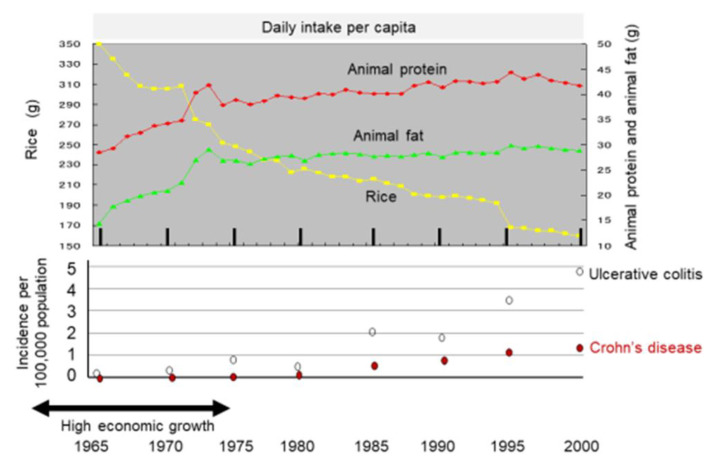
Chronological change in dietary intake and incidence of inflammatory bowel disease in Japan. The daily intake per capita of rice, animal protein, and animal fat is shown in the upper panel based on data for 35 years from 1965 to 2000 collected in the National Nutritional Survey. The incidence of ulcerative colitis and Crohn’s disease every 5 years is shown in the lower panel.

**Figure 5:**
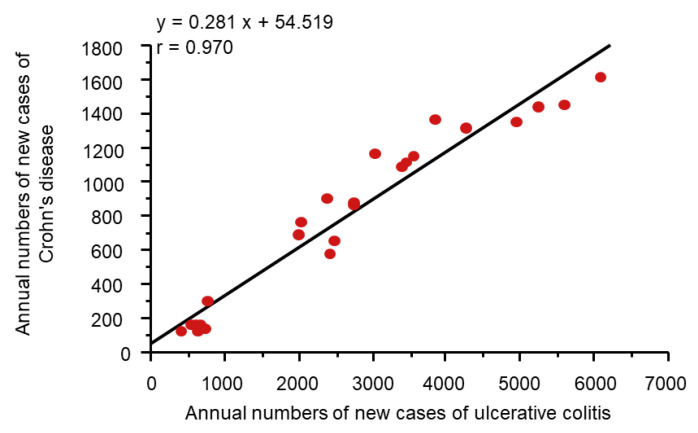
Correlation between Crohn’s disease and ulcerative colitis in the annual numbers of new cases in Japan. In Japan, the national registration of ulcerative colitis cases and Crohn’s disease cases started in 1975 and 1976, respectively. A scattergram was generated based on data for 24 years from 1977 to 2000. The linear regression formula and correlation coefficient are shown.

It might be more appropriate to look at dietary transition as a whole to evaluate dietary factors in IBD. Japan provides excellent conditions for analyses of dietary factors in IBD because the background of the Japanese population is rather homogeneous genetically and culturally. Increased incidence of IBD in association with dietary westernization was found in Japan (Figure 4) [14]. In addition, an extremely high correlation between the annual numbers of new cases of the respective diseases (r = 0.970) (Figure 5) indicates the presence of a common environmental factor in both UC and CD [14].

**Figure 6:**
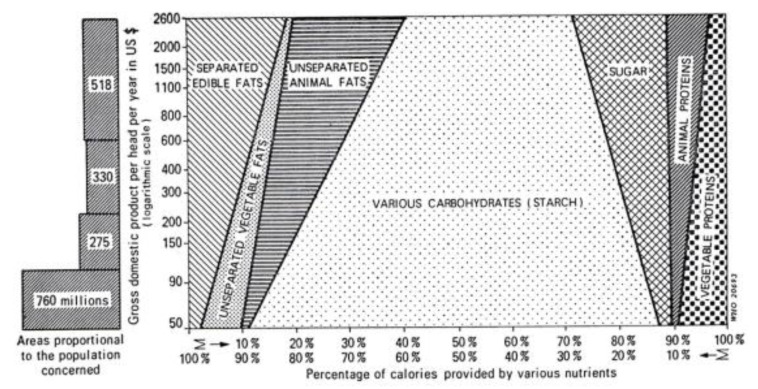
Calories derived from fats, carbohydrates, and proteins as a percent of total calories according to the income of the countries, data from [25].

**Figure 7:**
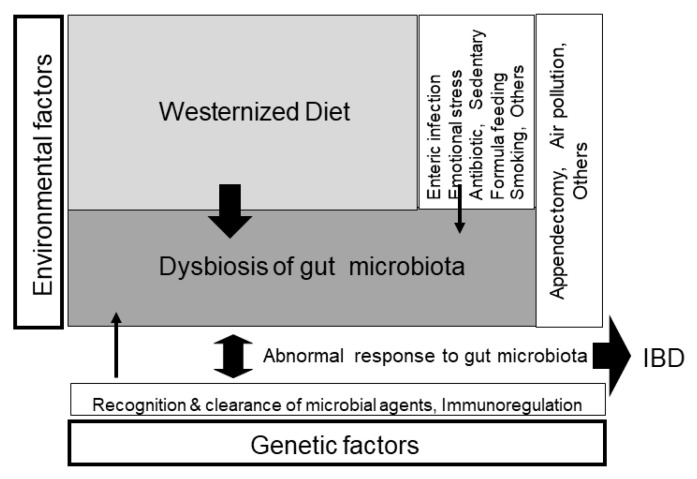
Schematic pathogenesis of inflammatory bowel disease (IBD). IBD occurs in genetically susceptible persons when triggered by environmental factors. The breadth of the arrow reflects the degree of the contributing role in the pathogenesis. The greatest environmental factor is gut dysbiosis (imbalance of gut microbiota), which is formed by a westernized diet, namely, westernized diet-associated gut dysbiosis [4] (with permission from the Permanente Federation).

There are at least two paths from traditional to current global diets. One is where it happens along with economic transition, as observed in developed countries (Figure 6) [25]. The other one is where it occurs along with the development of the food industry, where the population can afford cheap foods in developing or undeveloped countries [26]. As a result, current global consumption consists of an excess of unhealthy foods such as animal fat, animal protein, and sugar and a shortage of healthy foods such as vegetables and fruits [27,28]. This has caused the common diet-related chronic diseases we face: metabolic syndrome, diabetes mellitus, coronary heart disease, stroke, and non-alcoholic fatty liver disease. It recalls the proverb “we are what we eat” [29]. This means that the current omnivorous (westernized) diet is problematic and should be corrected. Not eating enough vegetables and fruits and excess consumption of animal fat, animal protein, and sugar are known risk factors for IBD [16,17,18,19,20,21]. Namely, the dietary pattern of the general population and IBD patients overlap. Therefore, we assert that dietary westernization is the most ubiquitous environmental factor in IBD (Figure 7) [3,4].

### 2.4. Onset of IBD during a Change in Dietary Habits towards Unhealthy Diets

Focusing on diet, we reported cases with new onset and relapse of IBD during a change in dietary habits toward unhealthy diets: UC during a low-carbohydrate weight-loss diet [30], pregnancy-onset UC [31,32], and relapse of UC in a patient with Takayasu arteritis [33]. 

The incidence of UC and CD is highest between the ages of 15 and 24 years old [34]. One typical pattern of onset of IBD in a 19-year-old male student is as follows [35]. He graduated high school in his hometown and entered a college in Tokyo. He moved from his hometown to Tokyo in April 2005. In November, 7 months after the move, loose stool/diarrhea appeared, followed by anal pain and soiled underpants. In March 2006, he was diagnosed with CD during a visit to his hometown. He started infliximab and a plant-based diet as first-line (IPF) therapy, which is described later. Remission was successfully induced. His plant-based diet score (PBDS), which was developed to evaluate adherence to a PBD for Japanese patients with IBD [36], is presented in Table 4. Eight items that seem to be preventive factors for IBD (vegetables, fruits, pulses, potatoes, rice, miso soup, green tea, and plain yogurt) were scored positively, while eight items that appear to be risk factors for IBD (meat, minced or processed meat, cheese/butter/margarine, sweets, soft drinks, alcohol, bread, and fish) were scored negatively. Scores of 5, 3, and 1 were given according to the frequency of consumption: every day, 3–5 times/wk, and 1–2 times/wk, respectively. The PBD score (PBDS) is the sum of plus and minus scores. A higher PBDS indicates a greater adherence to PBD. It is apparent that the quality of his diet had deteriorated (decreased PBDS from 14 to 6) during his life in Tokyo (Table 4). 

In these cases, patients were aware that they had changed their diets to unhealthy ones. We pointed out the patients’ risky dietary changes, and then the patients recognized the importance of diet. This seemed to result in their attention to diet and its contribution towards relapse prevention.

### 2.5. Current Westernized Diets vs. PBDs

The healthy reference diet recommended to the public suggests moderate animal food and sugar consumption and increased dietary fiber, namely vegetables and fruits [27,28]. Replacing our omnivorous westernized diet with a prudent (the healthy reference) diet has been recommended for decades [27,28,37,38]. Unfortunately, people have not necessarily followed the advice. There are many barriers to popularizing the healthy reference diet, including preferences for palatable diets, urbanization, availability of cheap, unhealthy foods, and lack of education on nutrition and lifestyle medicine in medical schools [38,39]. Moderating meat and animal protein and increasing daily consumption of vegetables and fruits are categorized as a PBD. PBDs are recommended to the public as a healthy diet to prevent common chronic diseases [27,28,40,41]. PBDs are more environmentally sustainable than meat-based diets in terms of greenhouse gas emissions, nitrogen and phosphorus pollution, biodiversity loss, and water and land use [27,41]. PBDs correspond to UN Sustainable Development Goals [27].

Recently, research has been unraveling the interplay between diet, gut microbiota, microbial metabolites, and health/disease [29,42,43,44,45,46]. The diseases extend beyond the confines of the gut (IBD) to various chronic diseases: obesity, diabetes mellitus, coronary artery disease, stroke, rheumatoid arthritis, cancer, psychiatric diseases, and others. We have coevolved with gut microbiota to exist in a symbiotic relationship. Westernized diets (high in fat, animal protein, and sugar, low in dietary fiber) decrease *Firmicutes* and increase *Bacteroidetes* at the phylum level: *Bacteroides* dominate at the species level. In contrast, PBDs (low in fat, animal protein, and sugar, high in dietary fiber) induce largely opposite changes. They increase *Firmicutes* and decrease *Bacteroidetes*: *Prevotella* dominates at the species level. In total, westernized diets tend to cause gut dysbiosis (reduced microbial diversity), while PBDs increase microbial diversity (symbiosis). *Firmicutes* include *Faecalibacterium* and *Roseburia*, which produce short-chain fatty acids by fermentation of dietary fiber. Short-chain fatty acids (butyrate, acetate, and propionate) are deeply involved in regulating host defense mechanisms [47]. Diverse beneficial effects of butyrate are well described: nutrition for gut epithelial cells, generation of colonic regulatory T-cells, a decrease of NF-kB-induced pro-inflammatory mediators (e.g., TNF-α, IL-6, IL-12, interferon γ), increase of anti-inflammatory mediators (e.g., IL-10), an increase of epithelial barrier function by antimicrobial peptide production, and enhanced mucus secretion via activation of G protein-coupled receptors 41, 43, and 109A [47,48]. Decreased production of short-chain fatty acids by westernized diets as compared with PBD is observed [29,45,46]. High consumption of animal protein results in increased production of ammonia, indoles, phenols, and hydrogen sulfide, which may be detrimental to our health [49]. Westernized diets are characterized by higher ratios of calories in fat with a lower ratio of carbohydrates [25]. Another characteristic of westernized diets is ultra-processed foods containing food additives (emulsifiers, thickeners, sweeteners), pesticides, and persistent organic pollutants. It has been recently reported that they increase the risk of IBD [50]. The sequence of phenomena leading towards a detrimental status as a result of a westernized diet is thought to be as follows: gut dysbiosis, disrupted intestinal barrier function, close contact with pathogens followed by translocation of pathogens, increased pro-inflammatory cytokines and oxidative stress, endotoxemia, and low subclinical inflammation [50,51,52,53]. It is of note that this path is thought to be common in IBD, obesity, and chronic diseases, including Type 2 diabetes mellitus, coronary heart disease, stroke, metabolic syndrome, and non-alcoholic fatty liver disease. Altogether, westernized diets are pro-inflammatory, and PBDs are anti-inflammatory [29,42,43,44,45,46]. These observations indicate that westernized diets increase susceptibility to IBD and other chronic diseases. They also show that refraining from westernized diets and replacing them with prudent diets is critically needed in IBD treatment.

## 3. Plant-Based Diets for IBD

### 3.1. Development of Plant-Based Diet for IBD

**Figure 8:**
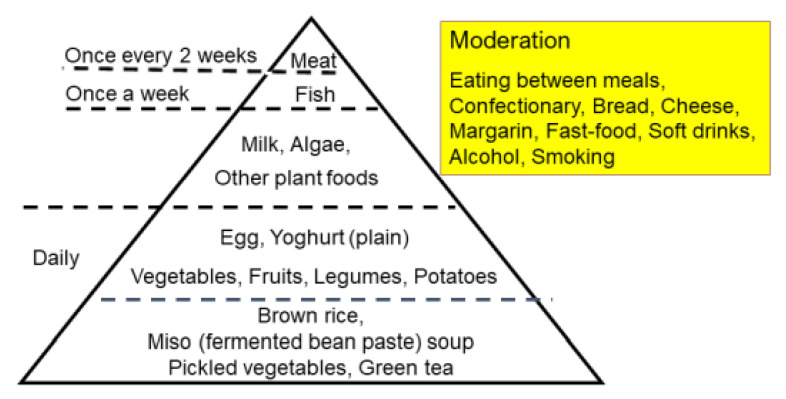
The semi-vegetarian-diet food guide pyramid.

**Figure 9:**
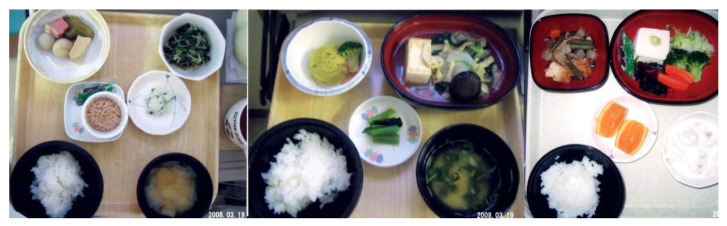
Semi-vegetarian diet (1400 kcal/day). From left to right: breakfast, lunch, and supper.

Epidemiology shows that an increase in IBD occurred after the dietary transition from a traditional diet to a westernized diet [14,54]. To counter westernized diets, we developed a PBD in the hope of increasing beneficial bacteria in the gut. A traditional diet is generally a plant-based diet. PBD incorporates many plant foods such as vegetables, fruits, beans, seeds, and nuts while minimizing animal foods (meat, fish), processed foods, and oils [40]. There are various types of PBD depending on the degree of animal food exclusion: vegan, lacto-ovo-vegetarian, semi-vegetarian, and pescatarian [40]. Our PBD is a lacto-ovo-vegetarian diet that allows for fish consumption once a week and meat every other week (Figure 8 and Figure 9) [5]. The proportions of protein, fat, and carbohydrates to total calories are 16.1 ± 0.5%, 18.6 ± 1.4%, and 66.1 ± 1.6%, respectively. It contains 32.4 ± 2.1 g of dietary fiber/2000 kcal (soluble dietary fiber 6.8 ± 0.7 g, insoluble dietary fiber 23.3 ± 1.6 g). Calories provided are about 30 kcal per kg standard body weight. There are no prohibited foods. It has been provided to all of our IBD inpatients since 2003. The plant-based diet score (PBDS) of our PBD is 35. On discharge, we advised patients on lifelong adherence to our PBD. Adherence to our PBD was 100% in inpatients and around 75% in outpatients [5]. Our lenient PBD does not result in micronutrient deficiency [41].

### 3.2. Indication of Infliximab and Plant-Based Diet as First-Line (IPF) Therapy

The natural history of most cases of CD is characterized by a disabling course [55,56,57], except for the 10–15% of lifelong relapse-free patients [58,59,60]. Clinical remission is defined as a Crohn’s Disease Activity Index (CDAI) score less than 150 in many studies [61], but this definition has some problems. A substantial portion of patients with symptoms of the active stage of CD who need treatment show CDAI < 150. In our experience, it was 18% (8/44) of patients [62]. Therefore, all patients with active symptom(s), irrespective of CDAI score, were advised to undergo hospitalization for possible IPF therapy. Between August 2003 and December 2015, 60 patients with active CD were admitted. The outcomes of 46 patients who were naïve to biologics were evaluated. We defined clinical remission as the disappearance of active symptoms of CD. The standard induction therapy with infliximab takes 6 weeks [63]. Therefore, we assessed remission at week 6 after the first infusion of infliximab [62].

Severe UC develops in 10–25% of UC patients [64]. It is a potentially life-threatening disease with a 1% mortality rate [64,65]. First-line therapy in the current guidelines is intravenous corticosteroids. Infliximab or cyclosporine is used as a second-line rescue treatment for patients unresponsive to corticosteroids [66]. Even though steroids are effective in the induction phase, steroid dependence or surgical intervention occurs in nearly half of such patients in the first year [67]. Because of this critical problem associated with steroids, we replaced steroids with infliximab together with PBD for severe UC [68]. Severe UC was defined by the Truelove and Witts criteria [64]. Patients were admitted and given the standard induction therapy with infliximab [68]. 

### 3.3. Protocol: IPF Therapy

**Figure 10:**
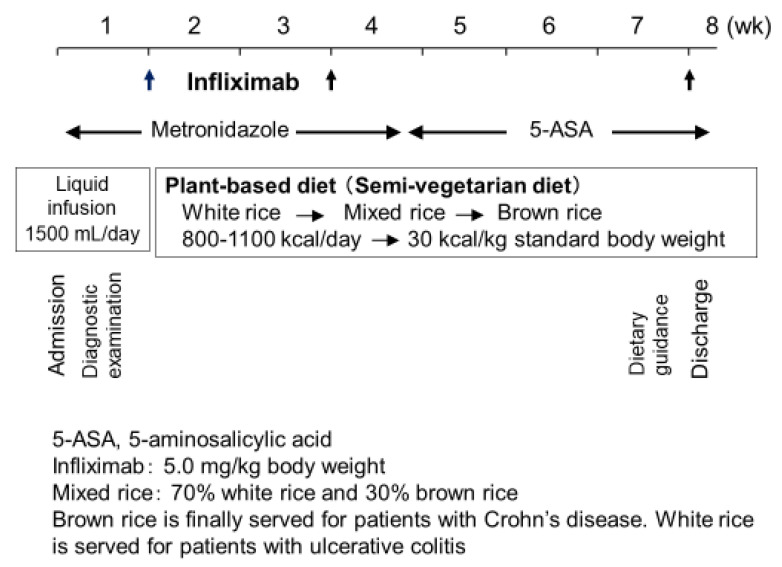
Protocol of infliximab and a plant-based diet as first-line (IPF) therapy.

As previously described, the protocol comprised standard induction therapy with infliximab combined with PBD (Figure 10) [5]. Briefly, metronidazole (750 mg/day) was given after admission. During morphological studies, patients received liquid infusion without meals to assess clinical types and intestinal stenosis. The length of liquid infusion varied from a few to several days, depending on the extent of previous outpatient studies before admission. Then, infliximab (Remicade, 5 mg/kg; Centocor, Malvern, PA, USA) was infused at weeks 0, 2, and 6 [63]. PBD was initiated on the same day of the infusion. Calories were gradually increased to a maximum of about 30 kcal per kg standard body weight. After about 1 month, metronidazole was switched to 5-aminosalicylic acids. After the third infusion of infliximab, patients were discharged. Patients who could not be admitted for the entire induction phase were discharged after the second infliximab infusion and readmitted for the third infusion [62].

### 3.4. Remission (Induction) Rates of IPF Therapy in CD

**Figure 11:**
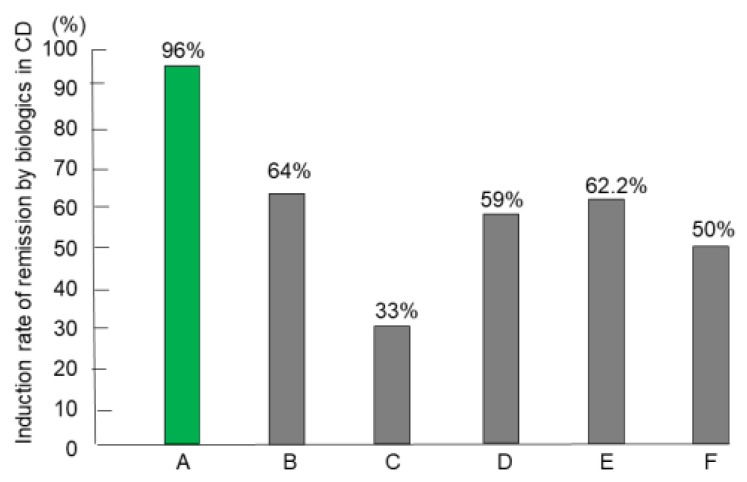
Induction rate of remission with biologics in patients with Crohn’s disease (CD) who were naïve to biologics. A: Infliximab and plant-based diet (*n* = 44) [62] B: Infliximab and azathioprine (*n* = 65) [70] C: Infliximab and azathioprine (*n* = 169) [71] D: Infliximab (*n* = 41) [72] E: Adalimumab (*n* = 45) [73] F: Ustekinumab (*n* = 191) [74].

Forty-six patients naïve to biologics comprised the intention-to-treat subset and underwent IPF therapy. However, two newly diagnosed patients, both men aged 21 years, with the stricture type, developed intestinal obstruction after the first infusion of infliximab. Consequently, they underwent surgery [69]. Therefore, 44 patients completed the protocol, comprising 24 newly diagnosed adults, 11 newly diagnosed children (≤18 years old), and nine relapsing adults. The mean disease duration of relapsing adults (92.8 months) was higher than the mean for newly diagnosed adults (8.8 months) or the mean for children (12.7 months) (*p* < 0.0001). More than half of the patients in all groups presented with perianal fistula(s) draining pus and/or anal tag(s). Five of 33 (15%) adults were current smokers who accepted the doctor’s advice and stopped smoking after admission. Eight patients had a CDAI score <150 (quiescent stage), seven 150–220 (mild-moderate), 19 220–450 (moderate-severe), and 10 >450 (severe/fulminant) [61]. Twenty-four patients with draining perianal fistulas experienced fistula closure within weeks 1 and 3. All 44 patients who completed the protocol achieved remission by week 6. Remission rates by the intention-to-treat and per-protocol analysis were 96% and 100%, respectively [62]. For the comparison, we tried to use the best, good, or representative results in the literature. Admitting that study designs differ from study to study, including subjects, therapeutic circumstances, and evaluation time for efficacy, the comparison shows what outcomes of therapy incorporating PBD are like (Figure 11) [62,70,71,72,73,74]. 

### 3.5. Remission (Induction) Rates of IPF Therapy in Severe UC

IPF therapy was administered in 17 severe cases of UC. The mean (SD) age was 43 (20) years. The number of initial episode cases, relapsing-remitting cases, and chronic continuous cases were 11, 4, and 2, respectively. The number of cases with extensive colitis and left-sided colitis was 13 and 4, respectively. The median (IQR) disease duration was 36 (11–103) months. The median (IQR) C-reactive protein level (normal range ≤ 0.3 mg/dL) and erythrocyte sedimentation rate were 4.5 (1.5–11.4) mg/dL and 54 (40–66) mm/hour, respectively.

The remission rate was 76% (13/17), and the colectomy rate was 6% (1/17) in the induction phase. C-reactive protein and erythrocyte sedimentation rates significantly decreased at week 6: 9.42 to 0.33 mg/dL and 59 to 17 mm/hour, respectively (*p* < 0.0001). At 1-year follow-up, the cumulative relapse rate was 25%, and there were no additional colectomy cases [68].

Remission rates have not been described for the most part when infliximab was used for severe UC as rescue therapy because the main concern was to avoid colectomy [64,66]. A systematic review showed that overall colectomy rates at 1 month, 3 months, and 1 year in severe colitis treated with standard infliximab rescue therapy were 10.6, 16.0, and 26.2%, respectively [66]. In our study, the colectomy rate was 6% at 1 month, which is lower than the 10.6% above. In addition, there was no increase in colectomy at 3 and 12 months. This indicates that incorporating PBD is beneficial in maintaining remission [68].

Rates of remission, incomplete remission, and colectomy with an intensive steroid regimen for severe UC were 40–58%, 24–26%, and 18–34%, respectively, in other studies [65,75,76]. Those in our study were 76, 12, and 6%, respectively: higher remission and lower colectomy rates. At 1-year follow-up, steroid dependence and additional colectomy accounted for approximately half of the patients treated with steroids in another study [67]. There was no case of steroid dependence or additional colectomy in our study. Therefore, short-term and medium-term outcomes with IPF therapy could be more effective for severe UC than intensive corticosteroid therapy.

**Figure 12:**
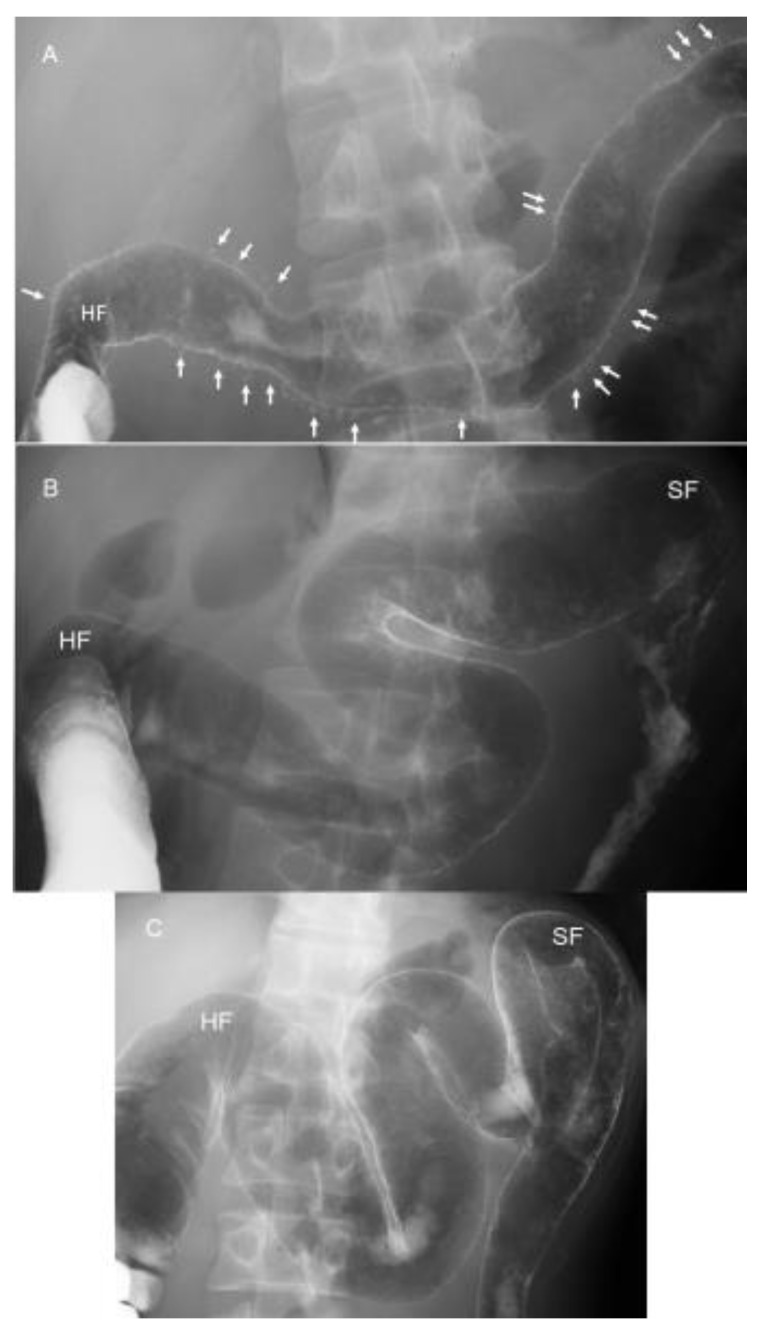
Roentgenograms of barium enema study during the induction phase in a 34-year-old man with severe ulcerative colitis (initial episode case) treated with infliximab and a plant-based diet as first-line (IPF) therapy. Numerous collar button ulcerations (arrows) are observed in the whole transverse colon 1 day before IPF therapy (**A**). They are resolved on the 13th day, 1 day before the second infliximab infusion (**B**). Further recovery with good distensibility is observed 1 day before the third infliximab infusion (**C**). HF, hepatic flexure; SF, splenic flexure.

IBD is a relapsing and remitting disease, and a morphological change of the large bowel occurs over time. Patients and physicians can easily understand the whole large bowel not through endoscopic photographs but by a roentgenogram of the bowel. Therefore, the authors perform a barium enema study when a diagnosis of UC is made and after treatment. The latter applies in severe or moderate cases. One of several advantages of IPF therapy is swift efficacy [68]. In one example, a 34-year-old man with diarrhea lasting 4 months and weight loss of 3 kg during the past 3 months visited us. He is one of 17 severe UC cases reported [68]. Barium enema examination just 1 day before IPF therapy disclosed numerous collar button ulcerations, a hallmark of severe UC [77] (Figure 12A). Swift recovery after IPF therapy prompted us to perform a barium enema study on the 13th day after the first infliximab infusion, just 1 day before the second infusion. Collar button signs were found to be resolved (Figure 12B). Further improvement was noted on the 41st day, 1 day before the third infusion (Figure 12C). He has been on sulfasalazine and in remission without relapse for 8 years. 

### 3.6. Absence of Nonresponders to Infliximab with IPF Therapy 

Primary nonresponders (PNRs) to biologics are well known and account for around 30% of patients in both CD and UC. Buhl et al. [78] clearly showed the poor prognosis of PNRs to infliximab in CD (9%, 33/353). More than half of such patients (58%) underwent surgery within 1 year after treatment failure, and a majority (71%) of those who avoided surgery were in the active stage despite a variety of medications at 1 year. The paper reassured us that successful remission induction was a prerequisite for a favorable prognosis. Jongsma et al. [72] compared the efficacy of first-line use of infliximab versus conventional exclusive enteral nutrition or prednisolone in therapy-naïve, new-onset pediatric patients with CD. They reconfirmed the superiority of infliximab therapy over conventional therapy in terms of clinical remission rate. However, the clinical remission rate at week 10 was 59% (Figure 11). It seems that even the first-line use of infliximab in therapy-naïve patients did not break the barrier of PNRs.

Many studies have attempted to identify the underlying mechanisms of and predict factors for PNRs. The effect of diet on the response to medications has recently begun to be recognized [79,80,81]. Aden K et al. [79] found that levels of butyrate and substrates involved in butyrate synthesis in fecal samples were significantly reduced in PNRs compared to responders both before and after treatment. Therefore, they stated that butyrate serves as a clinical marker for discrimination of responders and PNRs. Butyrate is a key substance in maintaining homeostasis in our body, including the gut, as stated above. Because butyrate is produced by bacterial fermentation of dietary fiber in our meals, the observation indicates that diet influences the effect of medication. The method of induction with infliximab is standardized. We administered the standard infliximab induction. The only apparent difference is the use of PBD in our study and the current omnivorous diet in other studies. PBD contains sufficient dietary fiber. Decreased consumption of dietary fiber is one of the characteristics of the current global diet. Therefore, it is likely that the absence or low rate of PNRs in our studies is due to PBD [82,83].

### 3.7. Relapse-Free Course in CD: Change of Natural History of CD

**Figure 13:**
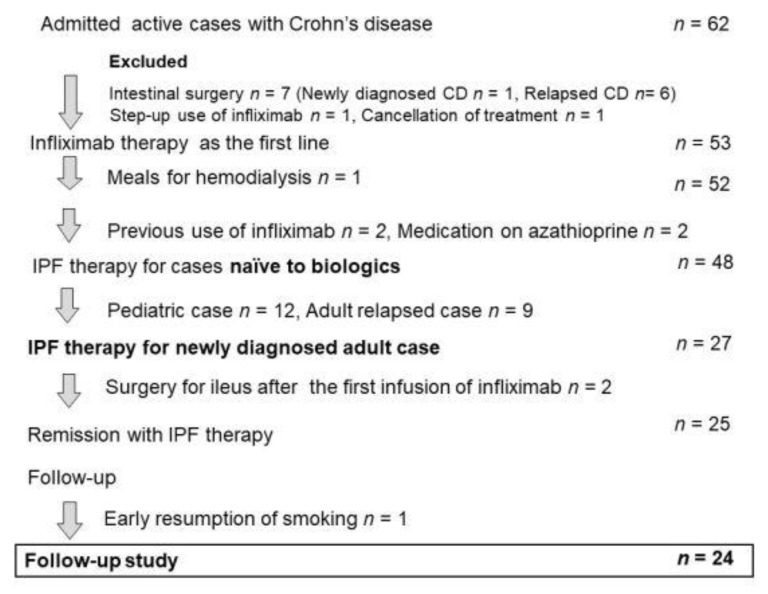
Enrollment of inpatients with active Crohn’s disease (CD) for IPF therapy. IPF therapy, infliximab, and a plant-based diet as first-line therapy.

**Figure 14:**
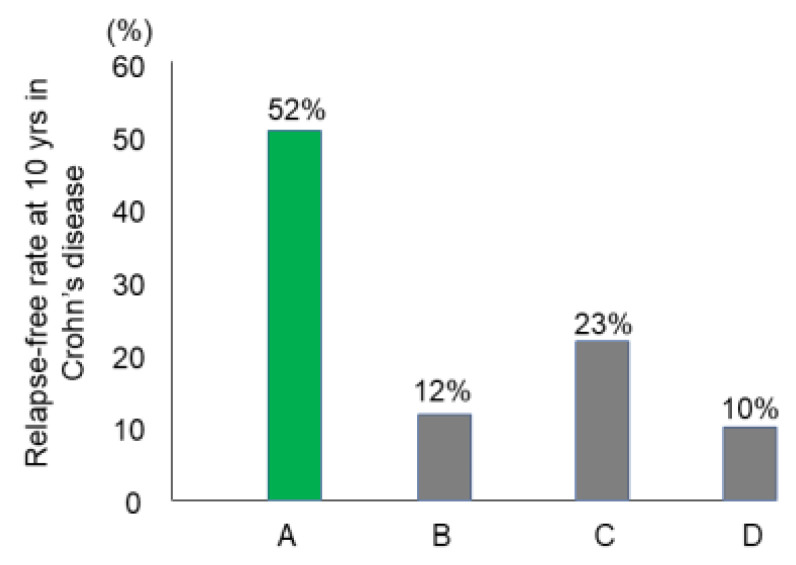
The relapse-free rate at 10 years after the first induction in Crohn’s disease. A: Report from Japan. Induction by infliximab and a plant-based diet as first-line therapy followed by the recommendation of adherence to a plant-based diet (*n* = 26) [84] B: Report from Denmark on induction by conventional therapy (*n* = 373) [58]. C: Report from seven European countries and Israel on induction by conventional therapy (*n* = 358) [59]. D: Report from Norway on induction by conventional therapy (*n* = 237) [60].

**Figure 15:**
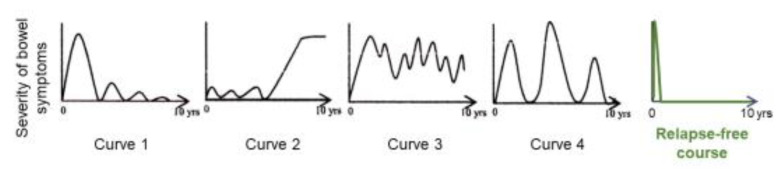
Graphic of clinical course in Crohn’s disease. Solberg et al. [60] presented four graphic clinical courses of CD: curve 1 (decrease in the severity of bowel symptoms), curve 2 (increase in the severity of bowel symptoms), curve 3 (chronic continuous bowel symptoms), and curve 4 (chronic relapsing bowel symptoms) (with permission from Elsevier). A relapse-free course was achieved in nearly half of the CD patients with infliximab and a plant-based diet as first-line (IPF) therapy (with permission from the Permanente Federation).

Preliminary follow-up studies indicated that the relapse rate was lower in newly diagnosed adult CD patients than in relapsed adults and newly diagnosed children [62]. We followed 24 newly diagnosed adult CD patients who were inducted into remission with IPF therapy (Figure 13) [84]. No biologics, immunosuppressants, or glucocorticoids were administered. The cumulative relapse-free rate was 52% at 10 years (Table 5, Figure 14) [58,59,60,84]. Solberg et al. [60] described four clinical courses in 191 CD patients in Norway: (1) decreasing severity of relapses, (2) increasing severity of relapses, (3) chronic continuous symptoms, and (4) chronic relapsing symptoms (Figure 15). Recently, Wintjens et al. [85] depicted six clinical courses in 432 patients in the Netherlands. Neither study described a relapse-free course. IPF therapy, on the other hand, created an unprecedented relapse-free course in nearly half of the patients with CD (Figure 15). 

The majority of CD patients experience relapse leading to four or six clinical courses based on the mode of relapse(s) above. A small portion of CD patients, however, do not experience relapse. Such relapse-free rates at 10 years were reported to be 10–23% (Figure 14). These studies were conducted before the use of biologics. To the best of our knowledge, this is the first report of long-term relapse-free rates in the biologic era. 

Reflecting the relapse-free course, the cumulative surgical rate at 5 and 10 years was 12% and 19%, respectively. Our surgical rates were lower than those reported (Table 5) [84,86,87,88,89,90,91].

### 3.8. Dietary Intervention, Lifestyle Medicine, and Self-Management Skills

It is obvious that the majority of diseases we face are chronic diseases (lifestyle diseases) due to an unhealthy lifestyle [92,93]. Therefore, incorporating a comprehensive healthy lifestyle in medicine based on pathophysiology, namely, lifestyle medicine, is fundamental for preventing and treating chronic diseases rather than medication or surgery alone. Bodai et al. [93] described the dramatic effect of and the need for lifestyle medicine in a variety of chronic diseases. Because dysbiosis of gut microbiota was found in a variety of common chronic diseases, and gut microbiota are formed by diet, dietary intervention focusing on restoring gut symbiosis is the current topic in various areas. Most of these studies set certain limited periods for adherence to a recommended diet [8]. This is a critical difference in dietary intervention studies between ours and others. We advise patients to continue a PBD as a life-long lifestyle choice. We tell patients that PBD is more important than medication for relapse prevention. This might explain why our patients showed significantly higher PBDS relative to baseline even several years after the introduction of PBD [84]. It seems that patients accepted PBD as a lifestyle.

Lifestyle changes, including dietary habits, are not easy [94,95,96]. It seems that the educational program [97] and PBD during hospitalization in our modality for IBD contributed to appreciation and alteration of lifestyle for health promotion. Many patients conveyed to us that their loose stool or constipation reverted to normal with greater adherence to PBD. Hospitalization seems to enhance the self-management skills of patients against relapse.

Dietary intervention should be comprehensive in IBD patients genetically predisposed to IBD. Japanese data indicated that both decreased consumption of rice and increased consumption of animal protein and animal fat were associated with an increase in IBD [14]. There will be a limitation to the mere exclusion of potentially untoward foodstuffs [98].

## 4. Concluding Remarks

There is heterogeneity between healthcare systems around the world. Therefore, the modality administered in our study cannot be immediately adapted to practice. Recognition of IBD as a lifestyle disease mainly mediated by our current diet will lead to a change in healthcare systems. Patients are always concerned about the side effects of medications. However, there is no worry about such side effects with PBD. Our modality fits the concept of the therapeutic goal in IBD, i.e., disease modification [99].

It is acknowledged that the evidence level of our single-group studies with small numbers of cases is low compared to conventional randomized controlled trials. By replacing a westernized diet with PBD, however, we consistently observed far better outcomes in both UC and CD in either the active or quiescent stage compared to the current standard [5,62,68,84,100,101]. PBDs are effective for all degrees of severity, from mild to severe, in both diseases. Our studies have yielded short- and mid-term outcomes but not long-term outcomes of more than 10 years. Based on such trials, it will take another decade to confirm the most suitable diet for IBD because not only the short-term but also the long-term effects of a suitable diet will need to be demonstrated. The content of PBD is consistent with the healthy reference diet. Considering the large number of patients suffering from inappropriate dietary guidance, we believe recommending PBD based on available evidence as the next best option is prudent, and it will greatly benefit IBD patients [102,103].

## Figures and Tables

**Table 1:** Top 10 research questions in the treatment of inflammatory bowel disease.

No	Content	Our Comment
1	What is the optimal treatment strategy considering efficacy, safety and cost effectiveness in IBD management?	IPF therapy is partly applcable.
2	What are the optimal markers combinations for stratification of patients?	None.
3	What role does diet have in the management of mildly active or inactive UC or CD to achieve normal daily activities and symptom control?	Diet plays a critical role.
4	How can pain be most effectively managed in people with IBD?	Relapse prevention by PBD particularly in CD.
5	What is an optimal treatment strategy for perianal CD?	IPF therapy is highly effective.
6	What is the best treatment for controlling diarrhoea and/or in continence symptoms?	Relapse prevention in cooperation with PBD.
7	What is the optimal dietary therapy?	PBD particularly in CD.
8	What is the association between IBD and fatigue and how should it be managed?	Relapse prevention in cooperation with PBD.
9	Does early surgery or later surgery for ileal CD result in better outcomes?	None.
10	Does influencing the gut microbiota influence the course of IBD?	Definitely indicated by literature.

CD, Crohn’s disease; IBD, inflammatory bowel disease; IPF, infliximab and plant-based diet as first-line therapy; PBD, plant-based diet; UC, ulcerative colitis.

**Table 2:** Environmental factors in inflammatory bowel disease.

Environmental Factor	Role in IBD			Exposure to the Majority of IBD Patients	Relevance to Gut Microbiota
	UC	CD	Mode of Role		
Lifestyle					
Smoking	P	R	Divergent in IBD	No	Yes
Diet				Yes	Yes
Animal protein	R	R	Identical in IBD	Yes	Yes
Dietary fiber	N	P		Yes	Yes
Tea or coffee	P	P	Identical in IBD	Yes	Unknown
Low levels of vitamin D	R	R	Identical in IBD	No	Unknown
Breast feeding	P	P	Identical in IBD	No	Yes
Pharmacological agents					
NSAID	R	R	Identical in IBD	No	Unknown
Antibiotics in childhood			Divergent among ethnic groups	No	Yes
Oral contraceptives	R	R	Identical in IBD	No	Unknown
Dipeptidyl peptidase-4 inhibitors	R	N		No	Unknown
Vaccination	N	N		Yes	Unknown
Appendectomy	P	R	Divergent in IBD	No	Unknown
Air pollution	R	R	Identical in IBD	No	Unknown

UC, ulcerative colitis; CD, Crohn’s disease; IBD, inflammatory bowel disease; P, protective factor; R, risk factor; N, neither protective nor risk factor: NSAID, non-steroidal anti-inflammatory drug.

**Table 3:** Case-control Studies on 37 foods for IBD onset in Japan.

	Crohn’s Disease (CD)	Ulcerative Colitis (UC)
Risky foods	Beef	
Excessive consumption	Greasy foods	
	Cheese	Cheese
	Butter	
	Sweets	Sweets
	Yoghurt (plain)	Yoghurt (plain)
	Natural fruit juice	
Preventive foods	Chinese cabbage	Liver
Shortage of consumption	Edible wild plants	Edible wild plants
	Tomato	Other fruits
	Mandarin orange	Mandarin orange
	Pickles	Pickles
	Green tea	Green tea
		Dried fishes
Identified to be neither risk	Salty foods, Pork, Chicken, Ham, Egg, Milk, Margarine, Fried food, Cabbage,	
nor preventive foods	Potato, Sauteed vegetables, Fresh fish, Mushroom, Processed fish paste,	
for both CD & UC	Spinach, Carrot, Tsukudani*, Cooked beans, Marine alga, Bean curd, Coffee	
Not identified to be risk or	Liver, Dried fishes, Other fruits	Beef, Greasy foods, Butter,
preventive factor for either		Natural fruit juice, Chinese cabbage,
CD or UC		Tomato

Painted foods are identical to both Crohn’s disease and ulcerative colitis. Tsukudani*: a preserved food made by cooking fish, kelp, animal meat, vegetables, etc. in sweetened soy sauce.

**Table 4:** Chronological change of plant-based diet score (PBDS).

	PBDS Scoring				PBDS in the Present Case			
Food Groups	Frequency of Consumption							
	Daily	3–5 times	1–2 times	Rarely	**A**	**B**	**C**	**D**
		/wk	/wk					
Vegetables	5	3	1	0	5	5	5	5
Fruits	5	3	1	0	1	0	5	0
Pulses	5	3	1	0	5	3	5	5
Potatoes/starches	5	3	1	0	3	3	5	5
Rice	5	3	1	0	5	5	5	5
Miso soup	5	3	1	0	5	5	5	5
Green tea	5	3	1	0	0	0	0 *	3
Yoghurt (plain)	5	3	1	0	1	0	5	1
Meat	−5	−3	−1	0	−1	−1	0	−1
Minced or processed meat	−5	−3	−1	0	−1	−1	0	0
Cheese/butter/margarine	−5	−3	−1	0	0	0	0	0
Sweets/ice cream/milk shake	−5	−3	−1	0	−3	−5	0	−1
Soft drinks (cola/ carbonated beverages/juice)	−5	−3	−1	0	−5	−5	0	−1
Alcohol	−5	−3	−1	0	0	0	0	0
Bread	−5	−3	−1	0	−1	−3	0	0
Fish	−2	−1	0	0	0	0	0	0
**Plant-based diet score (PBDS)**					**14**	**6**	**35**	**26**

A: before moving to Tokyo, B: after the moving, C: semi-vegetarian diet during the hospitalization, D: 2 years after the discharge * Green tea is recommended to drink at home but is not provided as a drink at the hospital.

**Table 5:** Literature review of relapse-free rate and surgical rate in adult Crohn’s disease.

Author	Country	Subjects & Year	Number of Cases	Cumulative Relapse-Free Rate		Cumulative Surgical Rate	
				5 Years	10 Years	5 Years	10 Years
Munkholm et al, 1995 [58]	Denmark	PBIC 1962–1987	373	22%	12%	n.d.	n.d.
Wolters et al, 2006 [59]	European countries	PBIC 1991–1993	358	31%	23%	21%	29%
Solberg et al, 2007 [60]	Norway	PBIC 1990–1994	237	15%	10%	27%	38%
Nguyen et al, 2011 [86]	Canada	2001–2008	1119			18%	n.a.
Frolkis et al, 2013 [87]	Meta-analysis	1990–	3239			28%	39%
		2000–	1193			24%	n.a.
Rungoe et al, 2014 [88]	Denmark	1995–2002	3718			27%	31%
		2003–2011	5552			20%	23%
Niewiadomski et al, 2015 [89]	Australia	PBIC 2007–2013	146			26%	n.a.
Kim et al, 2015 [90]	Korea	PBIC 2006–2012	11,267			9%	n.a.
Okada et al, 1994 [91]	Japan	1973–1988	58			29%	46%
Chiba et al, 2022 [84]	Japan	2003–2017	26	52%	52%	12%	19%

PBIC: population-based inception cohort, n.d.: not described, n.a.: not available (with permission from the Permanente Federation).

## Data Availability

The authors confirm that the data supporting the findings of this study are available within the article.

## Abbreviation

CD	Crohn’s disease
CRP	C-reactive protein
IBD	Inflammatory bowel disease
IPF	Infliximab and a plant-based diet as first-line
PBD	Plant-based diet
PBDS	Plant-based diet score
UC	Ulcerative colitis
